# Specific Action of Tissues, and Their Elements as Connected with the Living Body

**Published:** 1871-06

**Authors:** Daniel Lichty


CHICAGO
MEDICAL EXAMINER:

N. S. DAVIS, M.D., EDITOR.
F. H. DAVIS, M.D., ASSISTANT EDITOR.

VOL. XII. JUNE, 1871. NO. 6.

### Original Contributions.

### SPECIFIC ACTION OF TISSUES AND THEIR ELEMENTS AS CONNECTED WITH THE LIVING BODY.

BY DANIEL LICHTY, M.D.

The human organism presents itself as an aggregation of vital unities, every one of which manifests all the characteristics of life; and the separate and distinct peculiarities which these present, have given rise, among the students of physiology and pathology, to systems of research and theories of practice as numerous as there are sects in the Christian religion.

The ancients labored long under the delusion that the functions of the animal economy depended upon some *one* intrinsic force they variously denominated soul, archeus, or vital principle, which superintended the regular and perfect accomplishment of all the functions of living beings. Hippocrates studied carefully the effects and tendencies of such a force, and followed scrupulously all its indications. Galen led a school who believed that the elementary qualities of the *humors* presided over the functions of life. A third class, named Methodists, regarded solely the physical properties of the *solids*, and in particular perosity; attributing to the tissues only the two properties of contraction and expansion. Empirics there were, too, who wrongfully disclaimed physiological considerations, forgetting that in sciences as difficult and complicated as physiology and patholgy we must admit light from all the other sciences.

Had they been willing to aggregate these orders of forces, and say that the phenomena of the animal economy were produced *by them*, they would have approached nearer the right, and sooner aided in the perfection of our science. Then for awhile physiological processes were studied under different forms; the iatro-mechanics and iatro-mathematicians, versed in the calculations of the physical forces, assumed to explain the functions of the animal economy by the laws of mechanics; and the iatro-chemists took into consideration exclusively the mixture of chemical elements—the acid humors, gases, salts, and fermentations—each having its physiology, pathology, and therapeutics; and when their labors ceased, they left to science a great mass of theory, and to history the illustrious names of VanHelmont and Sylvius, the latter having attained commendable perfection in some of his aphorisms.

Thus the intricacies of physiology were being slowly unraveled, and the tissues were threatened to be robbed of their mysteries, when about the middle of the seventeenth century, Francis Glisson first recognized the phenomenon of *irritability;* he regarded this as a sufficient cause for *all* the phenomena of life, and supposed all the tissues possessed this force, though in different degrees, and proposed to divide it into natural, vital, and animal, accordingly as it was manifested by movements more or less apparent, with or without the concurrence of the will.

These ideas made but little impression on the medical world, and it needed the circumspect and ingenious experiments of Albert VonHaller, to elevate this hypothesis to a demonstrated fact. From this time physiology had an existence independent of physics and chemistry, and a *specific action* was demonstrated in the tissues; his researches, however, did not extend beyond the muscular tissue, and the unfinished work was left to *his* successors and *our* preceptors, who have with signal fidelity prosecuted it to a degree of perfection, so that the most recent researches still acknowledge a species of *excitation or irritation* necessary to prove vitality; and it is in fact the only criterion by which we can judge whether a tissue is alive or not; and our notion of death, or necrosis of a part, is based upon nothing more nor less than this; for we cannot exactly determine, by an anatomical examination, conducted either with or without a microscope, whether a nerve be alive or dead; nor can we judge the same of a muscle, since we find its structure perfectly preserved in parts that perished years ago. Czermak examined parts of mummies, and found in them tissues which were in a state of such perfect preservation, that the conclusion might have easily been arrived at, that the parts had been taken from a living body. Virchow found in a fœtus, which in a case of extra-uterine pregnancy, had lain thirty years in the body of its mother, the structure of the muscles as intact as if it had just been born at full time. Thus, however perfectly preserved the form of a part may be, without *irritability* or *activity* we have no right to suppose it possesses life. We now also recognize this property in the elements of every living tissue, whether it be muscular fibre, cell wall, continuous membrane, or ciliated epithelium; manifesting according to each tissue the particular processes of function, nutrition, or formation, the individual peculiarities of which, the space limited us will not allow us now to discuss. Hence we will proceed to that other property so nearly allied to this, that of *contraction*. This function was given to the medical world a little more than a century ago, and is still the subject of investigation.

Bichat demonstrated it to be a property that the *fluids* of the living body possess, and the coagulation of that essential fluid of the body—blood—is said to be due to contraction of a principle residing in it, variously denominated *fibrine* or *plasmine.*

But a short time ago, it was suppposed that certain forms of areolar or connective tissue were contractile. Leydig, Kolliker, and Virchow have each examined into this property, and the whole theory of contractility in the human body has been withdrawn within the limits of a substance in the febrils of muscles

—the red granular matter—which by polarized light can be seen to vary in appearance according to its degree of contraction, and which has, no doubt, as these investigations go to show, the property of contractility inherent in it.

These actions must have an exciting cause, and again we see the same tendencies presenting themselves as existed in the researches of the ancients.

Some referred it to the blood, as did one of the then leaders, Frederick Hoffman, who believed and said that "the blood excites the heart, which in its turn gives motion to the blood, thus giving the motor power to the functions of the animal economy;" but it will at once be seen that this is fallacious—for by this mechanism the cause produces an effect from which arises the reproduction of the cause itself, thus making his explanation turn in a vicious circle. Others regarded the nervous system the most important of all the organic apparatus—the one that first feels the impression of excitants, and transmits it to others; in a word, that gives impulsion to *all* the movements of the organism. But even the wide dissemination and the numerous connections which exist between the individual parts of the nervous system, are by no means calculated to show *it* to be the centre of all organic actions, besides, nervous action is spoken of very frequently, where it is now known that no nerves exist. It is true, we find in the nervous system definite elements which *serve* as centres of motion, but we do not find any single ganglion in which alone all movement in the end originates; we recognize a unity only in our own consciousness, for an anatomical or physiological unity, has at least, as yet, nowhere been seen by an anatomist, or demonstrated by a physiologist. These students forget, that *as* physiologists, they should endeavor to deduce the laws of living or organic forces from the direct observation of vital phenomena, in the same manner as philosophers and chemists deduce the laws of the general or organic forces from the observation of the phenomena in crude matter.

In *irritability* or *excitation* we recognize the first and only general action of living organisms. Contraction resides only in a certain element of the tissues, while the former belongs to every tissue. Yet, the numerous forms of activity manifested in and by the individual tissues of a body, lead us to believe, and numerous and able researches teach us, that they are not conducted by any one cause allotted to them from the beginning, but that in every one of them a certain excitation, a specific cause, is necessary for its production.

In studying pathological processes this will be still more apparent when we consider the specific excitants necessary to produce disturbances of a specific character—the specific diseases—so well-known, yet so *little* understood. Returning to physiology, it is made doubly apparent by considering the peculiarities of nutrition; here the function of the circulation of the blood, and the elements of the bloodvessels, manifest such varied and independent properties in the interchange of substance, that to deny a *specific action* would expose a lack of discernment and want of attention to these high functions. In the round of the circulation the blood is held by a continuous membrane, which in the capillary system is entirely devoid of porosity; though the experiments conducted recently at the Army Museum, where these membrane were magnified and remagnified by reflection several million times, seem to show pores; yet, if we speak of this as porosity, it can only be admitted in a physical sense as applying to really molecular interstices, which in the large multiplication employed in the above experiments, so dispersed these atoms as to show real interstices. A film of collodion cannot be more homogenous or continuous than this membrane; yet, there is inherent in the elements of its tissues a property that exercises a directly controlling influence upon the permeating powers of the fluids throughout its entire course; not, however, to such an extent as to control all the seeming peculiarities of leaving at one point much, at others little, and some none at all; for this *may* depend on the one hand *upon* the amount of pressure the blood is subjected to in certain localities, and on the other hand upon the special properties of the tissues. We cannot, by offering a part more blood, increase its nutrition; hyperiemia may occur and remain—the vessels gorged with blood for months—as is shown in section of the sympathetic nerve, and yet no appreciable nutritive changes occur. The frequent and continued congested condition of the genital organs induces no hypertrophy, either homologous or hetrologous; yet, for each particular tissue, be it nerve substance, muscular fibre, epithelium, or gland, there is in the blood a specific substance for which these tissues have a specific affinity, and in virtue of which their proper relations to each other and surrounding media is properly maintained. If it were not that these properties existed, and this membrane was of an indifferent character, the properties of Osmose and Dialysis would be restricted and controlled entirely by physical laws.

Gravitation of matter could only be governed by creating with active imagination centres at every convenient point, and at no time would the assurance be with us, that in the disturbance of some of these relations, an inundation might not extinguish the flame of life, or masses here and there accreting we should become half jelly-fish and half—What?

Another tissue possessing special action in a remarkable degree, is that of which the liver is constructed, and which performs its peculiar functions. The anatomical structure of the liver teaches that it is composed largely of vessels surrounding the hepatic cells, from which they are separated only by a thin layer of areolar or connective tissue. We are not allowed to suppose that the secretion of bile depends upon the peculiar distribution of the bloodvessels, for we find a similar network of a venous character too, in the lungs. Yet, the simple distribution of the capillaries of the portal vein about these cells, when in a condition of perfect health, afford us the phenomenon of not only secreting, but actually manufacturing a substance—bile—the matters which constitute it do not exist preformed in the blood; it is therefore apparent that the constituents of the bile arise not by a process of secretion, but by one of actual formation in the organ, and by the action of its tissues. Claude Bernard has thrown new and greater interest upon this question in his observations, showing that the property of producing sugar is also inherent in these elements; therefore, when we speak of the action of the liver in regard to both these important products, we can mean nothing but the special action of the individual elements of which it is composed. Let fat fill the cells, or they become extinct, or diseased, or their special properties in any way impaired, and neither the bloodvessels or the connective tissue can take up this action; it must unreservedly be attributed to a *specific action.*

The kidneys, too, have vessels afferent and efferent, with cells and connective tissue largely predominant in their composition; yet, their action is peculiar and essentially as different as their product. The membrane that separates the terminal branches of the pulmonary artery from the air cavities possesses properties by which oxygen is given to the blood-discs, and carbon dioxide with effete matter to the cavities; these properties may be dependent in part, as investigations now go to show, only upon the epithelium which line these cavities or walls; yet, we are compelled to admit, that even this argues more strongly than anything else could a *specific action* of these very epithelial cells, or of the basement membrane upon which they are situated, in which case *this* must possess the special property. If an independent action did not exist, then the functional disturbance or impairment of organs would not necessarily lead to such baneful results; for, vicarious action would be readily instituted, and the peculiar function of each not immediately arrested; but this vicarious substitution cannot be taken up by its own kind, simply, for these are located at many places, but each, by an *activity* of its *own*, whereby it is essentially characterized.

These nicely balanced and accurately adjusted conditions of the tissues and surrounding fluids of the living body, are brought about by *no* processes of a purely chemical character; if this is admitted, then we must admit strictly physical processes, and these we assert cannot exist where tissues possessing vital properties form the reservoirs which hold the fluids. That there are *affinities* existing between definite tissues and definite substances in virtue of which certain parts are enabled in a greater degree than others to attract certain substances from the neighboring blood, we will admit, but these very affinities are of such a specific character that they can attract and *select* from the surrounding media, not only the substances for their growth and repair, but actually transmit from atom to atom, or cell to cell, if you prefer, through matter of varying magnitude in the structures of the body to which vessels do not reach, and which are nourished only by this power of transmission residing in the elements of its tissues. These processes are apparent in the masses of tenden which have only bloodvessels at their peripheries, in the umbilical cord in which *no* vessels are given off from its veins or arteries into the Whartonian substance from within three or four lines of the umbilicus throughout its entire extent in the semi-lunar cartilages of the knee-joint, which are entirely detached from surrounding tissues, in the substance of the crystaline lens, and in the growth of cartilages in many forms; all these, and many other tissues, contain elements which possess this power in accordance with their several requirements; and, further, after having taken up this material, they are capable of subjecting it to further changes within themselves, in such a manner that they either derive therefrom new matter for their own repair and development, or that after these particles have imbibed this material, even decay may arise in their structure and their dissolution ensue, or an aggregation occur, which multiplying by direct ratio, speedily gives rise to pathological formations, homologous with the tissue in which it exists, or a foreign element finding its way into a tissue heterologous, formations can be rapidly produced by this action possessed by the element introduced; by pursuing this course of study, the early formation of many morbid enlargements will be made apparent.

By a similar independent action the various forms of degeneration pursue silently and surely their course. Fat insinuates itself into the muscles and prostrates the individual from want of action, or takes up its abode in the cellular interstices of connective tissue, and gives that roundness we admire in the figure of a lady, and which we always take as an indication of good living, unless it assumes the forms of obesity, polysarcia, or lipomata, when the condition of the individual is to be deplored. Ossific deposits occur distant from bone where no periosteum is known to exist; yet, it has previously been taught, that the presence of periosteum is necessary to the formation of bone. Tissues, then, of the living body, have residing in them elements by which they manifest their vitality, not only when acting conjointly with an organ, or through this with other organs, but a *special*, a *vital* action, by which their repair in perfect health is maintained, and by which an affinity is manifested for each other powerful enough for all the processes of life, yet so delicate in character that the quality of atmosphere we inhale, or even totally occult causes are capable of producing a perturbation of these elements, exciting morbid actions in the tissues, and consequent disease, which is only regulated by the amount and power of the disturbing influence, or controlling effect of therapeutic agents judiciously exhibited by a discerning physician. And as this presents therapeutics to our attention, we cannot pass it without a reference to the "specifics" now so frequently recognized by the profession.

Prof. Garretson, of the University Hospital of Edinburgh, said, a short time since, to his class during a clinic on erysipelas, "that the day was not far distant when the profession would recognize specific remedies as they now do specific diseases. And when we introduce these into our studies, *specific* actions will be sought for in every form of local disease, and the dyscrasie will have lost their terrors to the physician, while the tyro in our studies will sooner see that there is a truth and a certainty in them; and the chaos of darkness into which we are so often plunged, will be lighted by another lamp to guide the procession of our fraternity to the dignified position it has long sought among the sciences."

